# Effectiveness of digital self-care device for at risk drinking problems: focus on individuals at risk for alcohol-related issues

**DOI:** 10.3389/fpsyt.2025.1485940

**Published:** 2025-05-15

**Authors:** Yong Chan Jeong, Yong Jin Kim, Sungwon Roh, Eun Seon Seo, Hong Seok Oh, In Suk Lee, Eun Ji Lee, Hyeon Ji Cho, Sang-Kyu Lee

**Affiliations:** 1 College of Medicine, Hallym University, Chuncheon, Gangwon, Chuncheon, Republic of Korea; 2 Department of Social Welfare, Welfare and People Addiction Prevention Institute, Seoul, Seoul, Republic of Korea; 3 Department of Psychiatry, Hanyang University Seoul Hospital, Seoul, Seoul, Republic of Korea; 4 Department of Social Welfare, Integrated Addiction Management Support Center, Hwaseong, Gyeonggi, Hwaseong, Republic of Korea; 5 Department of Psychiatry, Konyang University Hospital, Daejeon, Daejeon, Republic of Korea; 6 Department of Nursing, Integrated Addiction Management Support Center, Suwon, Suwon, Republic of Korea; 7 Department of Counseling Psychology, Sahmyook University, Seoul, Republic of Korea; 8 Department of Psychiatry, Hallym University Medical Center, Chuncheon, Gangwon, Republic of Korea

**Keywords:** alcohol use disorder, digital self-care device, continuous days of sobriety, machine learning, ROC curve, multiple regression

## Abstract

**Aims:**

This study was conducted to verify the effectiveness of using digital self-care devices in reducing alcohol-related problems among high-risk alcohol users in community addiction-related institutions.

**Methods:**

Data were collected from 257 adults in Korea aged 18 and over (157 men and 60 women), examining their level of alcohol use disorder and the usage of digital self-care devices (such as the number of days alcohol consumption was logged, continuous days of sobriety, feeling, alcohol cravings, alcohol probability, etc.).

**Results:**

The results confirmed that the severity of alcohol use disorder significantly decreased before and after the use of digital self-care devices, as analyzed by a t-test (M = 5.239, SD = 10.121, t = 6.945, df = 179, P = .000***). Additionally, a machine learning analysis (random forest) was conducted to explore the factors that most influence the reduction in alcohol risk levels among participants. The analysis revealed that the factor “continuous days of sobriety” had the most significant impact on the reduction of alcohol risk levels. The predictive accuracy of this factor was demonstrated using an ROC curve (AUC = 0.724). Subsequently, a multiple regression analysis was conducted to explore the factors influencing continuous days of sobriety. The results indicated that age and the logging of sobriety days had a significant impact, with the logging of sobriety days emerging as the most influential factor.

**Conclusion:**

These results suggest that in reducing alcohol consumption and achieving successful sobriety, it may be more important to maintain continuous sobriety rather than the total number of sober days. Additionally, it is necessary to identify the key factors that help maintain continuous sobriety. Understanding which elements need to be fulfilled through digital self-care devices to sustain continuous sobriety is also essential.

**Clinical trial registration:**

https://cris.nih.go.kr/cris/search/detailSearch.do?search_lang=EM&focus=reset_12&search_page=M&pageSize=10M&page=undefinedM&seq=16267M&status=5M&seq_group=16267, identifier, KCT0005135.

## 1 Introduction

The mental health care system underwent significant changes due to COVID-19, and the effectiveness of telemedicine services had already been actively studied for a decade before the pandemic (1–3). The primary function of early telemedicine services was remote consultations and prescriptions. Regarding the anticipated concerns about patients’ attitudes toward receiving remote care, it was found that over 80% of participants who received consultations via video or phone had excellent or good attitudes toward the service (2). Based on these findings, as of 2021, 98% of psychiatrists in the United States were providing remote consultations to their patients (4). In addition to remote medical services, technologies such as digital therapeutic devices or self-management apps, which involve more active patient participation through web or app-based platforms, have been continuously developed. These advancements have further validated the necessity of telemedicine services (5, 6).

Most of the self-directed therapeutic devices developed are based on Cognitive Behavioral Therapy (CBT) as the primary treatment concept and offer services such as worksheets, self-assessment and monitoring, daily check-ins, and feedback on assessment results (7, 8). Additionally, recent studies have shown that structured psychological self-care using digital therapeutic devices for individuals with Alcohol Use Disorder (AUD) involved 8 weeks of patient-driven digital interventions, which demonstrated effectiveness in most participants (9).

According to a meta-analysis by Sliedrecht et al. (10), which reviewed literature from 2000 to 2019 on various relapse factors in patients with Alcohol Use Disorder (AUD), several key factors were identified as contributors to relapse. These include psychiatric comorbidities, addiction severity, alcohol cravings, negative emotions, use of other substances, and health and social factors. Notably, recent research has established a link between cravings and reduced alcohol consumption levels (11). Additionally, demographic factors have also been studied in relation to relapse and treatment outcomes, with job stability or marital stability being suggested as factors that contribute to successful treatment (12–14). Most studies focus on exploring the factors that lead to alcohol relapse or the development of Alcohol Use Disorder rather than therapeutic factors (15–18).

According to De Witte et al. (19), recent developments in the field of healthcare and welfare categorize remote healthcare services into three forms: (1) online management technologies, (2) user-driven expert interventions, and (3) new technology formats (e.g., VR, AR, wearables). Gan et al. (20) found that when comparing individuals who undergo digital therapy entirely in a self-directed manner to those who receive some level of intervention, the latter group showed slightly higher treatment efficacy. The device used in this study can be seen as a hybrid of (1) online management technologies and (2) user-driven expert interventions, primarily employed by patients visiting community centers but also including monthly in-person consultations. Specifically, the approach uses approximately 80% of (1) online management technologies and about 20% of (2) expert interventions, minimizing expert involvement while allowing users to self-direct most of the services.

Therefore, the purpose of this study is to verify the effectiveness of a pre-developed digital self-care device by recommending its use to patients who are at high risk for alcohol abuse, or who were previously patients, and who visit community centers. Additionally, assuming the effectiveness is validated, the study aims to identify the characteristics of factors that influence the reduction of alcohol use disorder risk, thereby deriving therapeutic factors for alcohol use disorders. The research questions of this study can be summarized as follows:

1. Verification of the effectiveness of the pre-developed digital self-care device.
2. Exploration of the characteristics of factors that influence the reduction of alcohol use disorder risk, assuming the effectiveness is validated.

## 2 Methods

### 2.1 Participant

**Figure 1:**
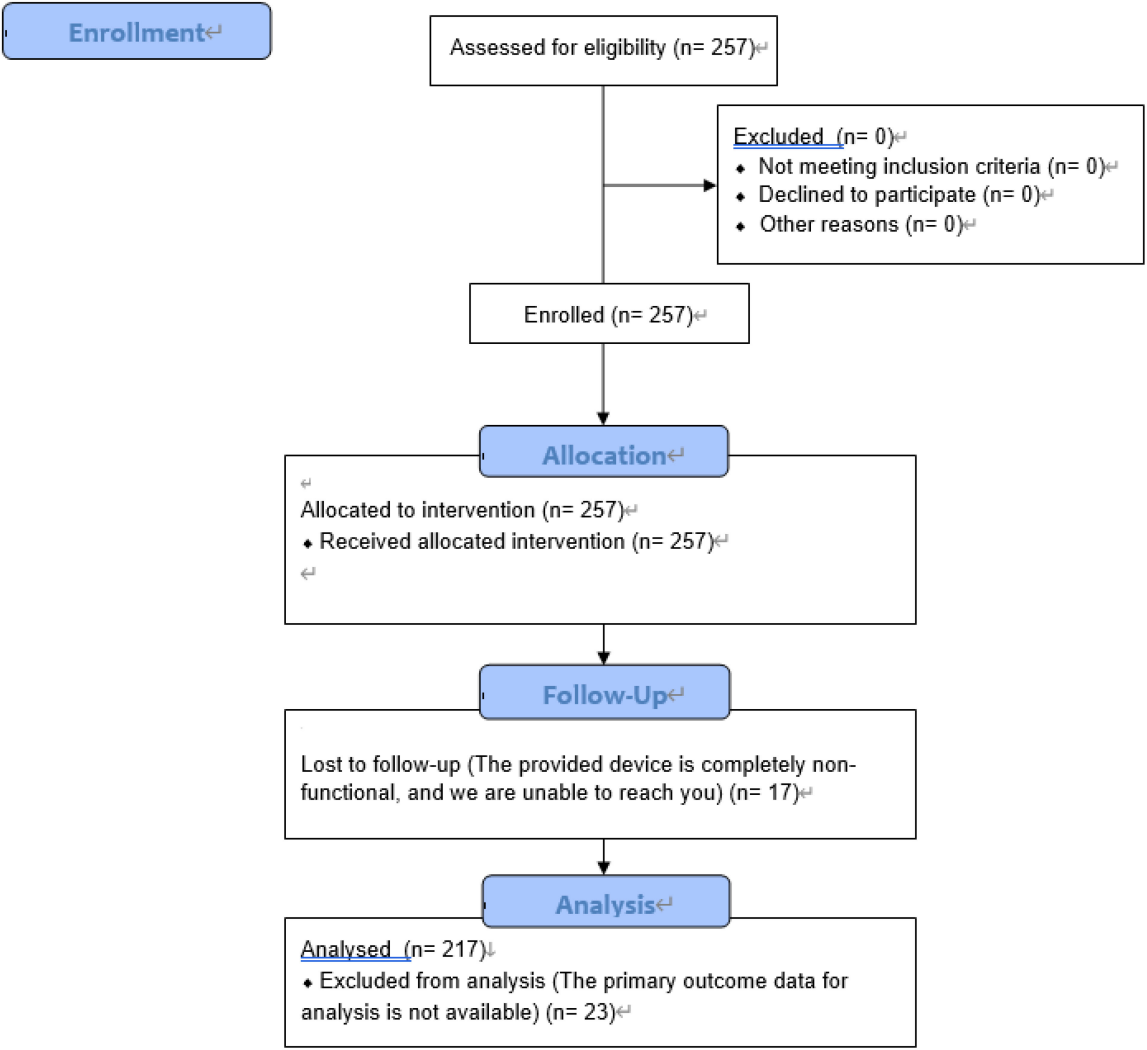
The clinical trial enrollment, assignment, tracking, and analysis process.

This study utilized data obtained from the clinical validation of a project requested by the National Center for Mental Health under the Ministry of Health and Welfare in Korea. The study aimed to verify the effectiveness of a digital self-care device and to explore predictive factors that could effectively reduce alcohol-related risks, leading to sobriety. A total of 257 participants were involved in the clinical validation, recruited from approximately 20 community addiction-related institutions across Korea. Participants were compensated with an in-kind reward equivalent to 100,000 KRW. The compensation was provided via direct bank transfer, with 50,000 KRW paid prior to the clinical trial and the remaining 50,000 KRW paid upon its completion. The majority of participants were aged between 40 and 60, and consent was obtained from them regarding participation in the clinical trial and the use of their information. After the validation process, participants were provided with a nominal incentive, and data from 40 individuals who dropped out during the study were excluded **Figure 1**. Consequently, the final sample consisted of 217 participants with demographic information as follows: the average age was 50 years (M = 50.51, SD = 12.63), with 157 males (72.4%) and 60 females (27.6%). The AUDIT-K was used as the measurement tool to identify the drinking status of participants, with the overall alcohol consumption levels categorized as follows: 38 participants (19%) were in the low-risk drinking group, 58 participants (25.3%) were in the high-risk drinking group, and 121 participants (55.8%) were in the alcohol use disorder group **Table 1**. Among the 40 dropouts, the demographic breakdown by gender showed 27 males and 13 females, with an average age of 63 years (M = 63.17, SD = 4.73). No significant differences were observed in other characteristics, but the average age was approximately 12.66 years higher than that of the 217 participants who completed the study.

**Table 1:** Demographic and clinical characteristics of participants (N = 217).

Characteristic	N (%) or Mean ± SD
Gender	
Male	154 (70.0%)
Female	60 (27.6%)
Missing value	3 (1.4%)
**Age (years)**	50.51 ± 12.63
Marital Status	
Single	65 (30.0%)
Married	82 (37.8%)
Other	66 (30.4%)
Missing value	4 (1.8%)
Education Level	
High school or below	138 (63.5%)
Bachelor’s degree	61 (28.1%)
Graduate degree	14 (6.4%)
Missing value	4 (1.8%)
Primary Diagnosis	
Low-risk drinking	38 (19%)
High-risk drinking	58 (25.3%)
Alcohol use disorder	121 (55.8%)

This study was approved by the Institutional Review Board (IRB) of the hospital to protect the rights, safety, and welfare of human subjects (IRB no. CHUNCHEON 2022-08-012). In addition, informed consent was obtained from participants to participate in the study and to use their information. Furthermore, this study utilized secondary analysis based on data collected from a clinical trial conducted in 2020, and the clinical trial registration number is as follows (Clinical Trial Registration Number (CRIS No.): KCT0005135).

### 2.2 Procedures

#### 2.2.1 Digital self-care application

The digital self-care device app used in this study is provided to individuals identified as high-risk for alcohol use by the Community Addiction Management Integrated Support Center. The app includes features such as a sobriety diary, self-assessment (for risk group classification based on the SBIRT stages), treatment schedule (to check the schedules of treatment programs registered in community centers), communication forums (recovery journal, sobriety communication meetings, and request-for-help communication), and support materials (card news, information about treatment facilities, and educational videos).

Upon the first launch, the app utilizes SBIRT and PPC to accurately assess the user’s condition and facilitate appropriate intervention. SBIRT (Screening, Brief Intervention, and Referral to Treatment) is an early intervention method designed to prevent issues in high-risk individuals from worsening by intervening early. PPC (Patient Placement Criteria) assesses six domains to determine the appropriate level of treatment based on the patient’s condition. Additionally, users can self-report and manage their condition through the sobriety diary feature, as shown in **Table 2**, and are required to use it consistently over a four-week period. The app also provides useful information through “online sobriety communication meetings” and resources like “card news and educational videos” to support sobriety efforts.

**Table 2:** Digital self-care measurement factors.

Factors	Measurement Method	Unit of Measure
Sobriety Status, Days of Sobriety	Yes/No	Yes = 1 No = 0
Continuous Sobriety Days	From the first Sober day to the next	
Feeling	Likert 1-7	1 = Very Bad 7 = Very Good
Craving Level		1 = Very High 7 = Very Low
Probability of Drinking		
Days of Sobriety Journaling	Sobriety Status, Today’s Feeling, Today’s Alcohol Carving, Likelihood of Drinking Today, Medication Status Completed sobriety diary at time of writing	

As a condition for using this device, the individual must first register for alcohol treatment at a community center. Once registered, the individual will receive an ID and password from their treatment provider to access the device. The user then progresses through the SBIRT stages within the device. If the user is classified as high-risk or above through the AUDIT-K within the SBIRT and consents to receive brief intervention, the PPC process will be initiated. The results of the PPC are then communicated to the user’s treatment provider, who uses this information to assist in creating an Individualized Service Plan (ISP) when the user visits the center.

In this study, only the results from the AUDIT-K within the SBIRT process and the measurements obtained through the sobriety diary were used for analysis, excluding the PPC results.

### 2.3 Measures

#### 2.3.1 Alcohol use disorder screening test

The Korean version of the Alcohol Use Disorders Identification Test (AUDIT-K) was used to determine the presence and severity of alcohol use among the participants in this study (21, 22). The AUDIT-K is a self-report measurement tool consisting of 10 items that assess an individual’s level of alcohol consumption (e.g., “How often do you have a drink containing alcohol?”). Each item evaluates factors such as the frequency and quantity of alcohol intake, as well as experiences within the past year. Higher total scores indicate a greater likelihood of alcohol use disorder. The Cronbach’s alpha for AUDIT-K is 0.92 (21), demonstrating high reliability.

#### 2.3.2 Digital self-care measurement factors

In this study, the digital self-care device allows for the monitoring of participants’ sobriety status. Participants can manage their condition by self-reporting their status through the sobriety diary feature within the device. The self-reporting measurement factors are composed of Yes/No questions and a 1-7 point Likert scale, as outlined in **Table 2**.

### 2.4 Data analysis

#### 2.4.1 Analysis method

In this study, SPSS Statistics version 27.0 was used to primarily analyze the effectiveness of the digital self-care device through t-tests. The analysis involved comparing the mean scores of the Alcohol Use Disorders Identification Test (AUDIT) administered before and after using the digital self-care device to determine the effect size. Additionally, to identify the main effects of the digital self-care device, the mean AUDIT scores of participants who fully engaged with the device’s content during the participation period were compared with those who did not engage at all.

To explore the factors that reduce the risk of alcohol use disorder, the R programming language was used with a machine learning technique, specifically the random forest algorithm. Finally, multiple regression analysis was conducted to analyze the characteristics that influence factors reducing the risk of alcohol use disorder. A total of 13 variables, including the dependent variable, the variables used in the analysis showed differences in scores due to differing reference points. By normalizing these scores into percentages, we standardized the contributions of the variables, allowing us to identify which variables had a greater influence on the dependent variable. The relevant variables are Sobriety Journaling, Total Sobriety, and Continuous Sobriety, with detailed information provided in **Table 3**. To demonstrate the predictive power of the identified factors through random forest analysis, a confusion matrix was used, with accuracy metrics serving as the validation measure. Precision and sensitivity were also presented to provide more specific values. Furthermore, to visually represent the predictive factors, feature importance was employed to indicate which factors had the greatest influence. To statistically express the precise predictive power of the explored factors, an ROC analysis was conducted to derive the AUC value. For the multiple regression analysis, the significance of the identified characteristics was defined using F, R-Square, B, β, and p-values.

**Table 3:** List of variables used in statistical analysis.

Analysis Methods	Variables Used	Variables Type
t-test	AUDIT-K Score(pre-post)	Continuous
	Overall Device Performance Rate(pre-post)	Categorical
Random Forest	Decreased AUDIT-K Score	Categorical
	Sex	Categorical
	Age	Continuous
	Marriage	Categorical
	Craving Level	Continuous
	Probability of Drinking	Continuous
	Feeling	Continuous
	Days of Sobriety Journaling	Continuous
	Rate of Sobriety Journaling	Categorical
	Total Sobriety Days	Continuous
	Total Sobriety Rate	Categorical
	Continuous Sobriety Days	Continuous
	Continuous Sobriety Rate	Categorical
Multi-Regression	Continuous Sobriety Days	Continuous
	Age	Continuous
	Sex	Categorical
	Today’s Feeling	Continuous
	Today’s Alcohol Carving	Continuous
	Likelihood of Drinking Today	Continuous
	Days of Sobriety Journaling	Continuous

#### 2.4.2 Missing value

When it comes to data analysis, there are various methods for handling missing values. In this study, it was determined that even after removing missing values, there would still be a sufficient sample size for conducting the analysis. Therefore, missing values were removed. As a result, the final sample size used for analysis was 175 participants, and this sample size was consistently applied across both the t-test and random forest analyses.

The 42 dropouts only participated in the pre-assessment during the 4-week clinical process and did not participate in the post-assessment, making accurate data analysis impossible. However, they were compensated for their participation in the study. Since there were no post-assessment results, they were excluded from the statistical analysis.

#### 2.4.3 Random forest

Random forest analysis is a method that improves predictive performance by combining multiple decision trees, a technique known as ensemble learning. It is primarily used for classification and regression tasks and enhances predictive accuracy by addressing the weaknesses of single models like decision trees. One key method random forest use to overcome the limitations of single models is “bagging” (Bootstrap Aggregating). In bagging, random subsets of training data are generated to create diverse models, thereby mitigating the weaknesses inherent in any single model. To reduce the correlation between variables, instead of considering all features as in a typical decision tree analysis, random forests consider only a randomly selected subset of features. This approach helps to reduce the risk of overfitting by limiting the model’s complexity and thus decreasing variance, which ultimately improves the generalization performance of the resulting model (23–25).

## 3 Results

### 3.1 Data analysis result

#### 3.1.1 The progression of alcohol use disorder severity

**Figure 2:**
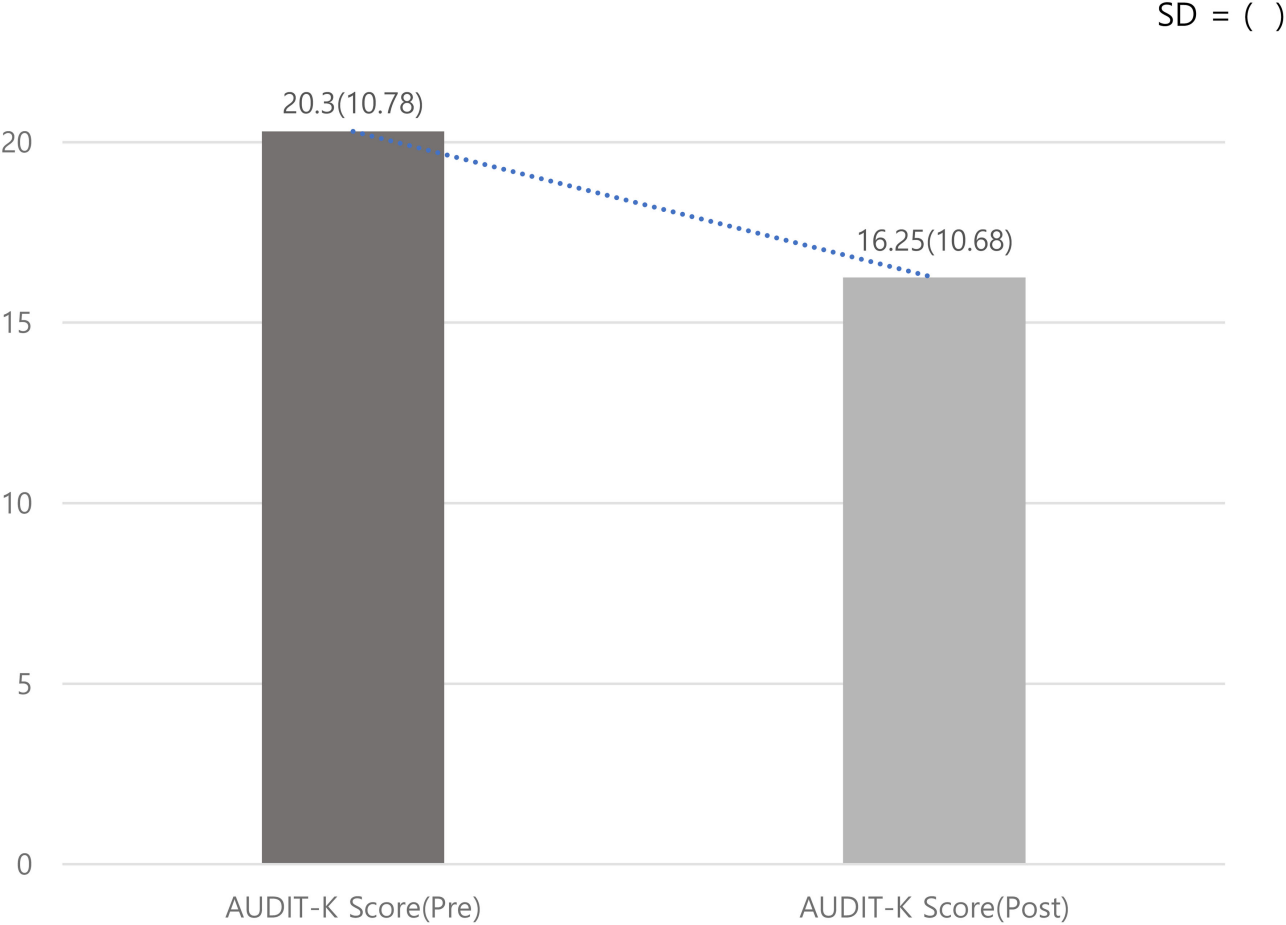
Comparing AUDIT-K Score pre-post Use of Digital Self-Care Devices. SD = ().

To analyze the results of this study, a paired-sample t-test was conducted. The results showed that the mean score of alcohol dependence severity (AUDIT-K) in the pre-intervention group was (M = 20.3), while the mean score in the post-intervention group was (M = 16.25) **Figure 2**. The mean difference between the pre- and post-intervention groups was (M = 5.239), (SD = 10.121), (t = 6.945), (P = .000***), indicating that the difference was statistically significant at the (p <.001***) level **Table 4**.

**Table 4:** AUDIT-K Score pre-post group t-test results.

AUDIT-K Score (pre-post)	Mean	SD	T	DF	P-value
	5.239	10.121	6.945	179	.000***

p-value < 0.001***.

**Figure 3:**
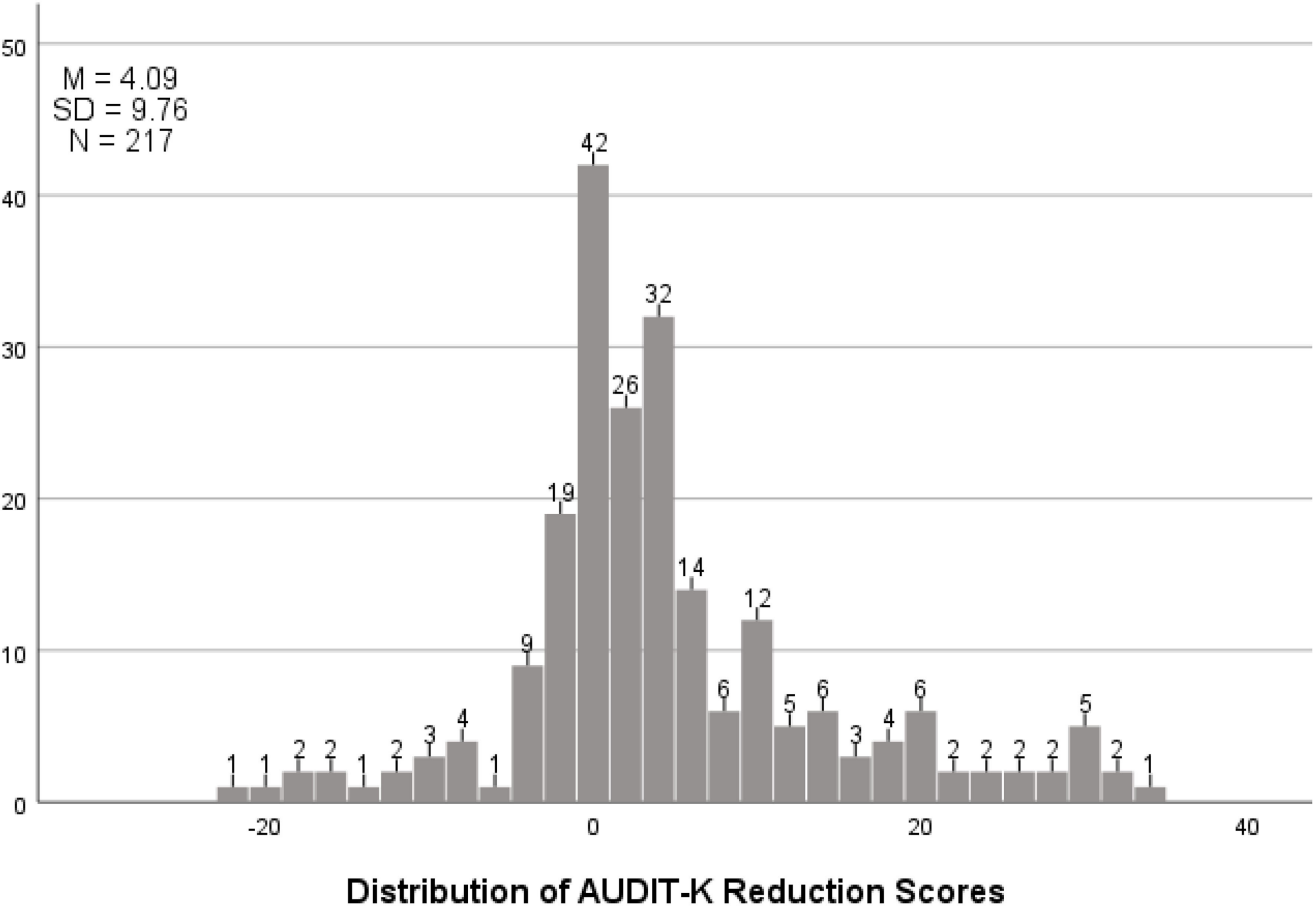
AUDIT-K score reduction distribution table.

The group used in the analysis was a single group, with the AUDIT-K scores measured before and after the intervention being used for comparison. A frequency analysis was conducted to compare the improvement scores based on the AUDIT-K scores, and the distribution of the overall scores was represented in a histogram. As a result, the AUDIT-K reduction score for all 217 participants was (M = 4.09), with a standard deviation (SD = 9.76) **Figure 3**. To provide more clarity, a frequency analysis was conducted by distinguishing between patients who experienced a reduction in their alcohol use disorder scores and those who did not. Among the 217 participants, N = 128 (59.0%) showed a reduction in their alcohol use disorder scores, while N = 86 (39.6%) did not. Missing data accounted for N = 3 (1.4%). As a result, the percentage of patients who experienced a reduction in their alcohol use disorder scores was 19.4% higher compared to those who did not **Table 5**.

**Table 5:** Reduction or non-reduction of AUDIT-K score(pre-post).

Reduction of AUDIT-K score(pre-post)	N (%) or Mean ± SD
Reduction	128 (59.0%)
Non-reduction	86 (39.6%)
Missing value	3 (1.4%)

**Figure 4:**
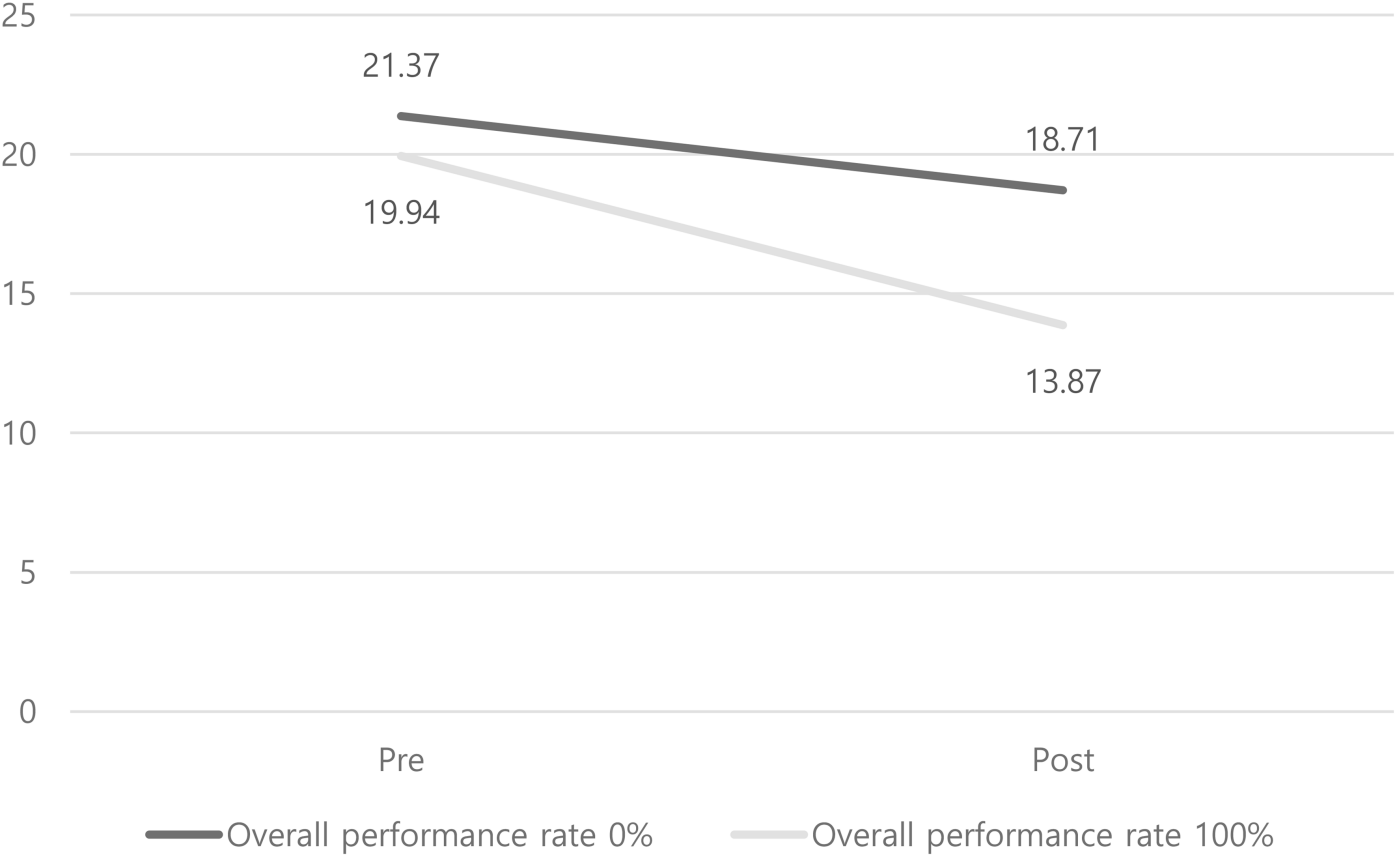
Compare average AUDIT-K score(pre-post) by overall performance rate.

Subsequently, based on the measured activity rate within the app over the study period, the 175 participants were divided into two groups: those who used the self-care device extensively (100% activity = 52 participants) and those who used it minimally (0% activity = 62 participants). Additional activity groups included 25% (28 participants), 50% (14 participants), and 75% (14 participants); however, due to extreme sample size differences, these groups were not included in the analysis. Given the short study duration of 4 weeks and the expectation that AUDIT-K scores might not show dramatic changes, we adopted a more conservative 99% significance level instead of the standard 95%. The results showed that the pre- and post-intervention mean scores for the 0% activity group were (M = 21.37) and (M = 18.71), respectively, while those for the 100% activity group were (M = 19.94) and (M = 13.87), respectively. When examining the significance of the mean differences, the 0% activity group showed (M = 2.651), (SD = 10.052), (t = 2.093), (P = .040), indicating that the p-value was not statistically significant. In contrast, the 100% activity group showed (M = 6.075), (SD = 10.181), (t = 4.344), (P = .001***), revealing a statistically significant difference at the (p <.001***) level **Table 6**, **Figure 4**.

**Table 6:** Frequency analysis results by performance rate.

Type	Group(N)	Mean (SD)	Group(N)	Mean(SD)
Age	Performance Rate 0% (62)	52.5(1.169)	Performance Rate 100% (52)	46.5(12.871)
Feel		4.81(1.37)		5.31(1.16)
Craving		5.21(1.49)		6.11(1.02)
Probability		5.34(1.70)		6.34(.88)

Type	Group(N)	Mode(N)	Group(N)	Mode(N)
Marital Status	Performance Rate 0% (62)	1 = marriage(27)	Performance Rate 100% (52)	2 = unmarried(23)

To gain a more detailed understanding of the observed results, a frequency analysis was conducted to examine the characteristics of each group. Differences were found in variables such as ‘age,’ ‘marital status,’ ‘mood,’ ‘alcohol craving,’ and ‘likelihood of drinking.’ In terms of age, the 100% activity group had a relatively lower average age compared to the 0% activity group, with a significant difference in standard deviation as well **Table 7**. Additionally, the 0% activity group scored lower on mood, craving, and likelihood of drinking compared to the 100% activity group. These scales were measured using a 1–7 point Likert scale, with reverse scoring applied to the craving and likelihood of drinking variables, meaning that a score closer to 7 indicated a better state for all variables. Finally, regarding marital status, the 0% activity group had a higher proportion of married individuals compared to the 100% activity group.

**Table 7:** Compare average AUDIT-K score(pre-post) by overall activity rate.

	Mean	SD	T	DF	P-value	95%CI	
						Lower	Upper
Overall Activity Rate 0%	2.651	10.052	2.093	62	.040	1.119	5.182
Overall Activity Rate 100%	6.075	10.181	4.344	52	.001***	3.269	8.882

p-value < 0.001***.

After conducting a frequency analysis, a chi-square test was performed to determine whether there were mean differences between the characteristics of the 0% and 100% activity groups. However, no significant differences were found between the characteristics.

#### 3.1.2 Exploration of predictive factors

**Figure 5:**
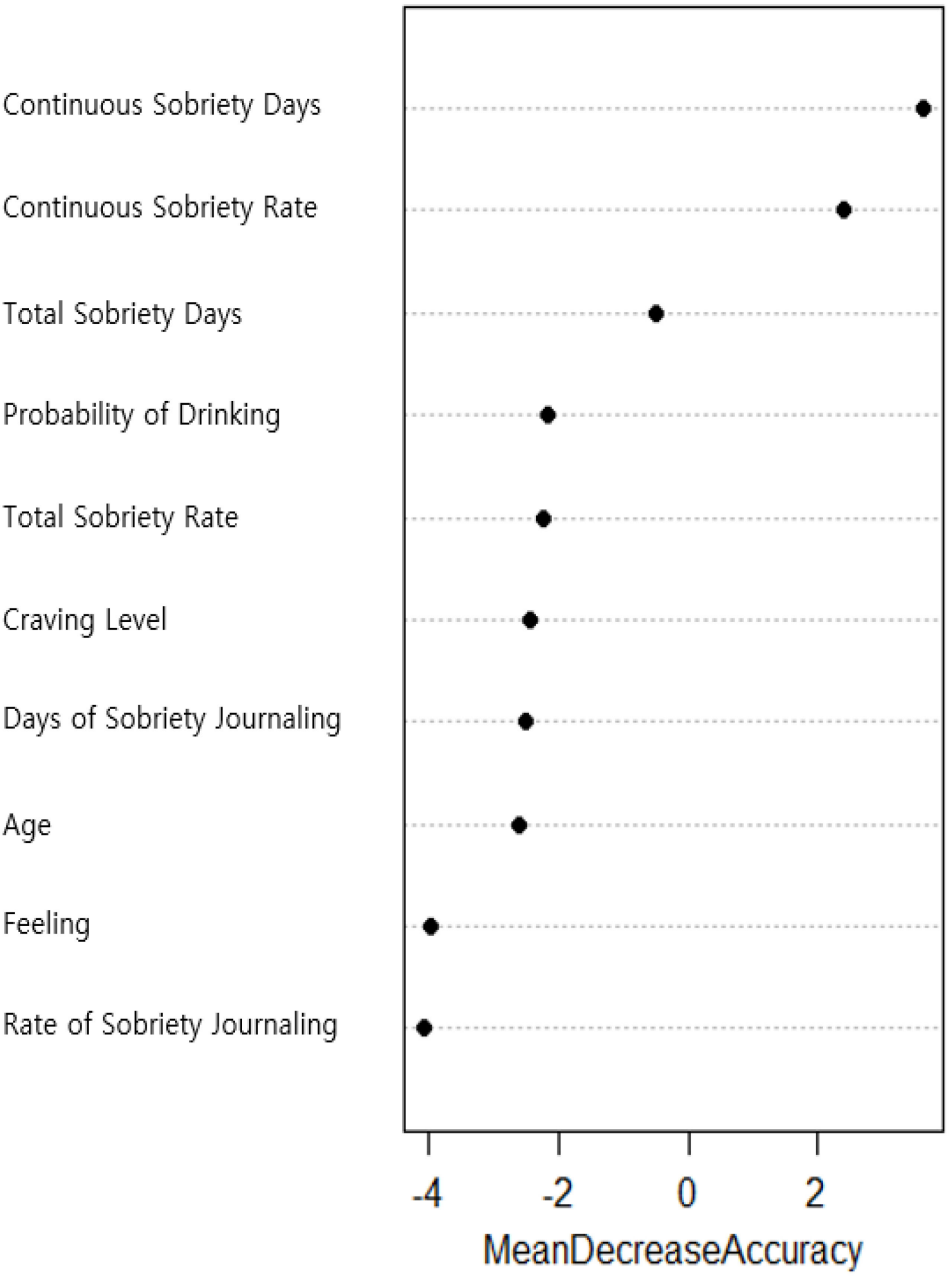
Predictor feature importance results.

Earlier, the effectiveness of the digital self-care tool was verified through a paired-sample t-test. Following this, factors and characteristics collected through the digital self-care tool and questionnaires were organized, and a representative machine learning technique, random forest, was used to analyze which variables actually influence the “reduction in alcohol dependence severity”. The analysis revealed that “consecutive days of sobriety” had the most significant impact on the “reduction in alcohol risk.” Additionally, according to the feature importance in the random forest analysis, apart from “consecutive days of sobriety” and “participation rate in consecutive sobriety,” no other variables significantly influenced the level of alcohol risk reduction **Figure 5**. Interestingly, the result that “consecutive days of sobriety” had a greater impact on reducing alcohol risk than “total days of sobriety” is somewhat unusual and will be discussed in more detail in the conclusion and discussion sections.

**Figure 6:**
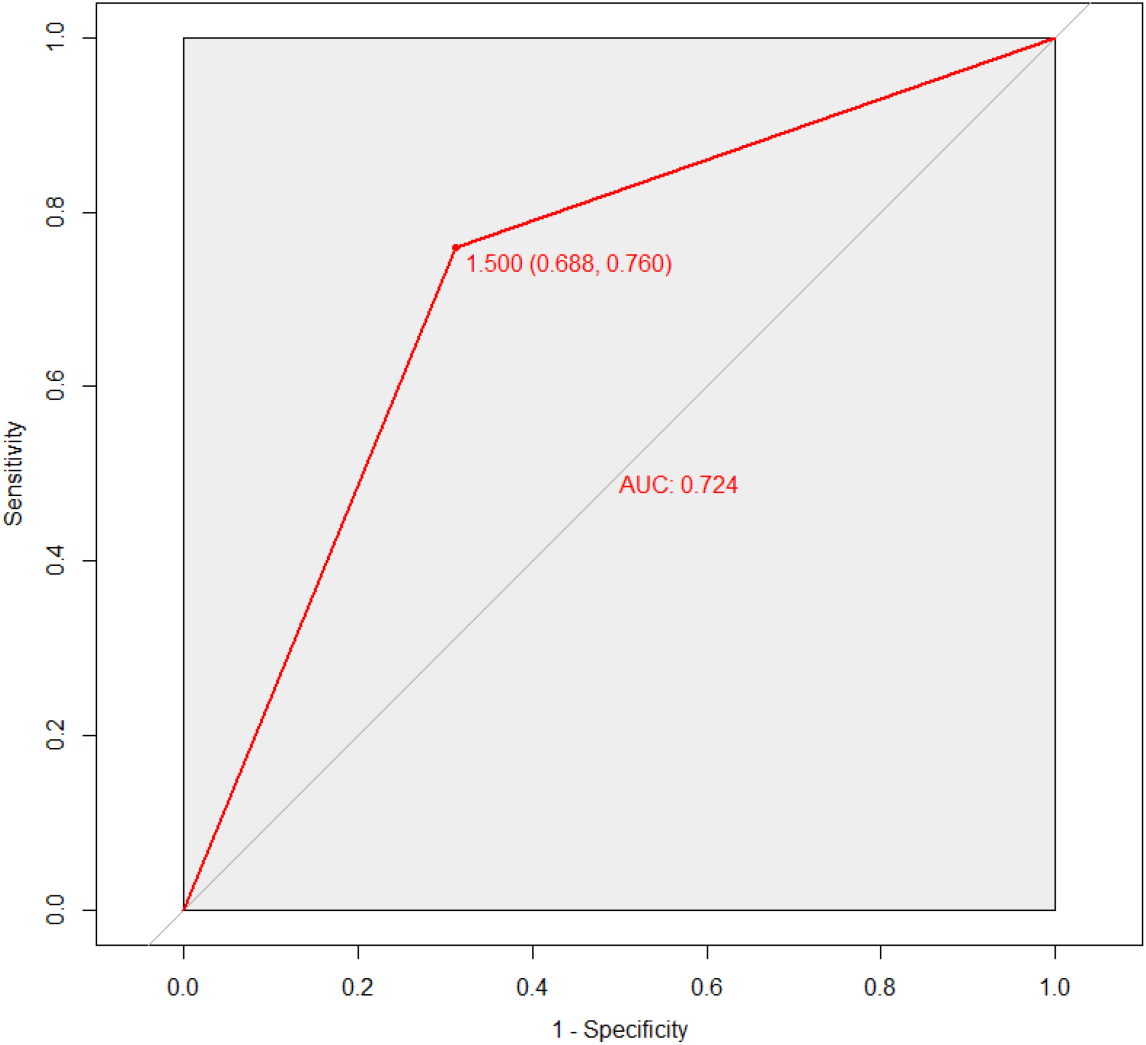
Result of ROC curve for random forest model performance.

Furthermore, the prediction accuracy was found to be 0.7317, which is a highly significant value. For a prediction model to be considered accurate and reliable, the confusion matrix should show high TP (True Positive) and TN (True Negative) values, with low FP (False Positive) and FN (False Negative) (**Table 8**). In this analysis, the confusion matrix showed TP = 11, TN = 19, FP = 6, and FN = 5, indicating very good predictive results. Additionally, the precision and sensitivity of the model were also strong, with precision at 0.7600 and sensitivity at 0.6875, suggesting that the model is stable. To visually assess the model’s performance, an ROC Curve was utilized, and the results indicated that the AUC (Area Under the Curve) value was 0.724. This confirms that the model has quite good predictive performance **Figure 6**.

**Table 8:** Confusion matrix.

Reference(Real World)			
Prediction		Yes	No
	Yes	True Positive(TP)	False Positive(FP)
	No	False Negative(FN)	True Negative(TN)
Accuracy: $\frac{TP+TN}{TP+FP+FN+TN}$			

Reference(Real World)			
Prediction		Alcohol Risk Reduction (Yes)	Alcohol Risk Reduction(No)
	Alcohol Risk Reduction (Yes)	11	6
	Alcohol Risk Reduction (No)	5	19
Accuracy: 0.7317			
Specificity: 0.7600			
Sensitivity: 0.6875			

#### 3.1.3 Predictive factors for continuous days of sobriety

As previously identified through random forest analysis, consecutive days of sobriety were found to be a key factor in reducing alcohol risk levels. To explore which factors collected through the digital self-care device influence consecutive days of sobriety, a multiple regression analysis was conducted. The stepwise selection method was chosen for this analysis.

The analysis results demonstrated that the regression model was appropriate, with F = 68.319, (p <.001). The adjusted R-squared value was (*R*^2^ = .699), indicating that the model explained 69.9% of the variance in consecutive days of sobriety. Among the predictor variables used in the analysis, age was found to have a significant effect on consecutive days of sobriety, with B = -.135, (p <.05). Since the coefficient B is negative, it indicates that for every one-year increase in age, consecutive days of sobriety decrease by 0.135 days. Additionally, the number of days the sobriety diary was completed (Create Diary) was shown to have a significant positive effect on consecutive days of sobriety, with B = .822, (p <.001). This means that for every additional day the sobriety diary was completed, consecutive days of sobriety increased by 0.822 days. Other variables did not have a significant impact on consecutive days of sobriety **Table 9**.

**Table 9:** Factors influencing continuous sobriety days.

Variables	Non-Standardized Coefficients		Standardized Coefficients	t(p)	*TOL*	*VIF*
	B	SE	*β*			
(Constant)	-8.963	5.361		-1.672		
Age	-.135	.066	-.088	-2.060*	.937	1.067
Sex	-1.015	1.832	-.023	-.554	.924	1.082
Feel	-1.365	.844	-.088	-1.617	.984	1.016
Carving	2.150	1.255	.148	1.714	.581	1.722
Failure	1.873	1.058	.141	1.770	.233	4.283
Create Dairy	.822	.047	.750	17.339***	.272	3.674
*F(p)*	68.319***					
adj.*R*^2^	.699					
Durbin Watson	2.101					

^*^p<.05, ^**^p<.01, ^***^p<.001.

When comparing which variable had a greater influence on consecutive days of sobriety between age and the number of days the sobriety diary was completed, the standardized coefficients (β) were examined. The results showed that age had a (β = -.088), while the number of days the sobriety diary was completed had a (β = .750), indicating that the completion of the sobriety diary had a relatively higher influence on consecutive days of sobriety compared to age.

## 4 Discussion

### 4.1 Discussion

This study was conducted to verify the effectiveness of self-care among individuals at high risk for alcohol use and to identify factors that reduce alcohol risk levels. While research related to remote digital medical devices and self-care is actively ongoing worldwide (7–9), there appears to be a lack of studies, like this one, that utilize self-care devices through nationwide institutional collaboration within a country. Additionally, while many previous studies have focused on identifying factors that contribute to the onset or relapse of alcohol use disorder (15–18), this study aimed to explore whether factors that reduce alcohol risk levels could be identified through self-care and to determine which specific characteristics could contribute to this reduction.

As numerous remote digital therapeutic devices and self-care tools are being developed, proving their effectiveness has often been challenging. However, in this study, participants who regularly visited community institutions (e.g., addiction centers, mental health welfare centers) for alcohol treatment monitoring (using the SBIRT framework) and simultaneously used a remote digital self-care device demonstrated significant effectiveness. This aligns with previous research findings, which suggest that self-care approaches are more effective when combined with some level of expert intervention rather than relying solely on a fully self-directed digital therapeutic process (20). Moreover, unlike other self-care devices used in different studies, the self-care tool in this research emphasized helping users manage their schedules and providing indicators to assess their own condition. It also indirectly indicated that their case managers were overseeing their progress. Given this approach, the study found that for individuals at a high risk of alcohol use disorder—not necessarily those already suffering from it—basic monitoring combined with self-care can effectively reduce alcohol risk levels and maintain longer periods of sobriety. This approach marks a departure from previous studies, which often focused more on therapeutic interventions. In conclusion, the results of this study provide significant clinical implications for the reliability, validity, and effectiveness of remote digital self-care devices. The study not only highlights differences from previous research but also offers substantial insights for future studies in this area.

Additionally, while many studies have focused on identifying the factors that contribute to the onset of alcohol use disorder, the significance of this study lies in its exploration of key factors that can reduce alcohol use disorder. The results indicate that consecutive days of sobriety had the most substantial impact on reducing alcohol risk levels, which is quite a unique finding when compared to the total number of sober days. This is particularly noteworthy because, among participants using the device, those with a high total number of sober days did not necessarily experience significant reductions in alcohol risk levels compared to those with high consecutive days of sobriety. In some cases, despite having a high total number of sober days, participants did not see substantial changes in their risk levels, or their risk levels remained unchanged. Therefore, the only independent predictor that effectively reduces alcohol risk levels can be considered as “consecutive days of sobriety.” This finding aligns with previous research (26), which suggests that the effectiveness of alcohol treatment increases when sobriety is maintained consecutively. Future studies should consider utilizing the LASSO method instead of regression analysis to evaluate more accurate predictive variables. Additionally, extending the clinical period and increasing the number of evaluations should be considered to improve the assessment outcomes.

In this study, participants with a 100% activity rate showed lower cravings and a reduced likelihood of drinking compared to those with a 0% activity rate. Simultaneously, their alcohol risk levels also decreased more than those in the 0% activity group. This suggests that as the consecutive period of sobriety increases, it likely has a greater impact on reducing cravings and the probability of drinking. Furthermore, the total number of sober days simply represents the cumulative days of sobriety over four weeks, meaning it might not reflect continuous sobriety. This raises the possibility that some participants may have relapsed during the period. For instance, a participant who had a total of 20 sober days over four weeks but drank during the first two weeks and then remained sober for the last two weeks might still exhibit higher levels of craving, a greater likelihood of drinking, and higher alcohol risk levels compared to someone who maintained continuous sobriety throughout the period.

### 4.2 Limitations

Despite the conclusions and discussions drawn from this study, there are three significant limitations to consider. The first limitation concerns the measurement tools used. Specifically, the tools within the digital self-care device relied on a 1-7 point Likert scale in a self-report format, rather than using established psychological measurement instruments. This approach has inherent limitations, as it depends heavily on the honesty of the participants due to the single-item nature of the questions. The use of only one item per measure makes it challenging to fully establish the reliability and validity of the participants’ reported states. However, to mitigate these limitations, the study instructed social workers and mental health professionals at each community institution to conduct weekly monitoring. Additionally, participants were required to visit the centers weekly to review their status with professionals. Even though the more comprehensive measurement tools were included in the device, the high number of items may have caused participants to feel burdened by the assessment process. This is a chronic issue with digital therapeutic and self-care devices, and finding solutions to this problem will require further research in future studies.

The second limitation is the clinical duration. Although over 20 official public institutions operated by the Korean government participated in this study, the clinical period was limited to just four weeks. This short duration made it impossible to follow up on the participants’ conditions after the clinical trial, preventing an assessment of the long-term effectiveness of the self-care device. Despite this limitation, the satisfaction levels of the patients who used the device during the four-week period were high, and the study demonstrated actual effectiveness. This suggests that the self-care device used in this study may play an important role in helping participants maintain sobriety and prevent relapse, even within a relatively short time frame.

The final limitation lies in the insufficient exploration of key variables related to treating alcohol use disorder. For instance, if the study had delved deeper into major variables such as the reasons for patients seeking treatment for alcohol use disorder—namely, “treatment motivation”—it might have identified additional key predictors that contribute to reducing alcohol risk scores. While this study focused on evaluating the effectiveness of the self-care device, future research should aim to identify various factors to explore the key variables most influential in treating alcohol use disorder. This shift in focus could provide deeper insights and enhance the understanding of impactful factors in treatment.

### 4.3 Conclusion

In conclusion, the findings of this study suggest that conducting a satisfaction survey for the digital self-care device developed in this research could be beneficial. By identifying any discomforts or areas in need of improvement and then updating the device accordingly, it could be re-released with enhanced functionality. Such improvements would enable individuals at risk of alcohol use disorders to manage themselves more effectively, potentially alleviating socio-economic issues in South Korea and reducing social costs.

Lastly, this study holds significant importance as the first to utilize a digital self-care device across community institutions nationwide in South Korea. It marks a crucial step forward in the development and advancement of research on digital healthcare and self-care devices in the country. The study involved approximately 40% of addiction-related community institutions in South Korea, with more than 70 addiction and mental health experts collaborating. Additionally, with a minimum of 10 participants recruited from each region, the results of this study are considered to be generalizable within the South Korean context.

As a follow-up to this study, two approaches can be considered to examine the sustainability of the effects. First, tracking the participants of this clinical trial to assess how long the effects persist. Another approach would be to extend the duration of the clinical trial to 8 or 12 weeks to evaluate the sustainability of the effects.

## Data Availability

The original contributions presented in the study are included in the article/Supplementary Material. Further inquiries can be directed to the corresponding author.

## References

[1] Hubley S Lynch SB Schneck C Thomas M Shore J . Review of key telepsychiatry outcomes. World J Psychiatry. (2016) 6:269. doi: 10.5498/wjp.v6.i2.269 27354970 PMC4919267

[2] Guinart D Marcy P Hauser M Dwyer M Kane JM . Patient attitudes toward telepsychiatry during the COVID-19 pandemic: a nationwide, multisite survey. JMIR Ment Health. (2020) 7:e24761. doi: 10.2196/24761 33302254 PMC7758084

[3] Hensel J Graham R Isaak C Ahmed N Sareen J Bolton J . A Novel Emergency Telepsychiatry Program in a Canadian Urban Setting: Identifying and Addressing Perceived Barriers for Successful Implementation: Un nouveau programme de télépsychiatrie d’urgence en milieu urbain canadien: Identifier et aborder les obstacles perçus d’une mise en œuvre réussie. Can J Psychiatry. (2020) 65:559–67. doi: 10.1177/0706743719900465 PMC7492888 31969011

[4] American Psychiatric Association . Psychiatrists use of telepsychiatry during COVID-19 public health emergency. (2020).

[5] Tofighi B Chemi C Ruiz-Valcarcel J Hein P Hu L . Smartphone apps targeting alcohol and illicit substance use: systematic search in in commercial app stores and critical content analysis. JMIR mHealth uHealth. (2019) 7:e11831. doi: 10.2196/11831 31008713 PMC6658280

[6] Gratzer D Torous J Lam RW Patten SB Kutcher S Chan S . Our digital moment: innovations and opportunities in digital mental health care. Can J Psychiatry. (2021) 66:5–8. doi: 10.1177/0706743720937833 32603188 PMC7890581

[7] Chen H Rodriguez MA Qian M Kishimoto T Lin M Berger T . Predictors of treatment outcomes and adherence in internet-based cognitive behavioral therapy for social anxiety in China. Behavioural and Cognitive Psychotherapy. (2020) 48(3):291–303. doi: 10.1017/S1352465819000730 31928568

[8] Haug NA Morimoto EE Lembke A . Online mutual-help intervention for reducing heavy alcohol use. J Addictive Dis. (2020) 38:241–9. doi: 10.1080/10550887.2020.1747331 32314667

[9] Kraepelien M Sundström C Johansson M Ivanova E . Digital psychological self-care for problematic alcohol use: feasibility of a new clinical concept. BJPsych Open. (2023) 9:e91. doi: 10.1192/bjo.2023.73 37222099 PMC10228278

[10] Sliedrecht W de Waart R Witkiewitz K Roozen HG . Alcohol use disorder relapse factors: A systematic review. Psychiatry research. (2019) 278:97–115. 10.1016/j.psychres.2019.05.038 31174033

[11] Tuchman FR Hallgren KA Richards DK Aldridge A Anton RK Aubin HJ . Reductions in WHO risk drinking levels correlate with alcohol craving among individuals with alcohol use disorder. Alcohol: Clin Exp Res. (2024) 48(2):420–429. doi: 10.1111/acer.15257 PMC10922776 38149364

[12] Walton MA Blow FC Bingham CR Chermack ST . Individual and social/environmental predictors of alcohol and drug use 2 years following substance abuse treatment. Addictive Behav. (2003) 28:627–42. doi: 10.1016/S0306-4603(01)00284-2 12726780

[13] Bottlender M Soyka M . Efficacy of an intensive outpatient rehabilitation program in alcoholism: predictors of outcome 6 months after treatment. Eur Addict Res. (2005) 11:132–7. doi: 10.1159/000085548 15990430

[14] Walter M Gerhard U Duersteler-MacFarland KM Weijers HG Boening J Wiesbeck GA . Social factors but not stress-coping styles predict relapse in detoxified alcoholics. Neuropsychobiology. (2007) 54:100–6. doi: 10.1159/000096991 17108710

[15] Brady KT Sonne SC . The role of stress in alcohol use, alcoholism treatment, and relapse. Alcohol Res Health. (1999) 23:263. 10890823 PMC6760383

[16] Stillman MA Sutcliff J . Predictors of relapse in alcohol use disorder: Identifying individuals most vulnerable to relapse. Addict Subst Abuse. (2020) 1:3–8. doi: 10.46439/addiction.1.002

[17] Dandaba M Serra W Harika-Germaneau G Silvain C Langbour N Solinas M . Predicting relapse in patients with severe alcohol use disorder: The role of alcohol insight and implicit alcohol associations. Addictive Behav. (2020) 107:106433. doi: 10.1016/j.addbeh.2020.106433 32289744

[18] Sliedrecht W Roozen HG Witkiewitz K de Waart R Dom G . The association between impulsivity and relapse in patients with alcohol use disorder: a literature review. Alcohol Alcoholism. (2021) 56:637–50. doi: 10.1093/alcalc/agaa132 33382416

[19] De Witte NA Joris S Van Assche E Van Daele T . Technological and digital interventions for mental health and wellbeing: an overview of systematic reviews. Front Digital Health. (2021) 3:754337. doi: 10.3389/fdgth.2021.754337 PMC8732948 35005695

[20] Gan DZ McGillivray L Han J Christensen H Torok M . Effect of engagement with digital interventions on mental health outcomes: a systematic review and meta-analysis. Frontiers in digital health. (2021) 3:764079. 10.3389/fdgth.2021.764079 PMC8599127 34806079

[21] Lee BO Lee CH Lee PG Choi MJ Namkoong K . Development of the Korean version of the Alcohol Use Disorders Identification Test (AUDIT): A reliability and validity study. Addict Psychiatry. (2000) 4:83–92.

[22] World Health Organization . AUDIT: the Alcohol Use Disorders Identification Test: guidelines for use in primary health care(2001). Available online at: https://www.who.int/publications/i/item/WHO-MSD-MSB-01.6a (Accessed April 17, 2024).

[23] Breiman L . Random forests. Mach Learn. (2001) 45:5–32. doi: 10.1023/A:1010933404324

[24] Cutler DR Edwards TC Beard KH Cutler A Hess KT Gibson J Lawler JJ . Random forests for classification in ecology. Ecology. (2007) 88(11):2783–92. doi: 10.1890/07-0539.1 18051647

[25] Biau G Scornet E . A random forest guided tour. Test. (2016) 25:197–227. doi: 10.1007/s11749-016-0481-7

[26] Dunn KE Harrison JA Leoutsakos JM Han D Strain EC . Continuous abstinence during early alcohol treatment is significantly associated with positive treatment outcomes, independent of duration of abstinence. Alcohol Alcoholism. (2017) 52:72–9. doi: 10.1093/alcalc/agw059 PMC5169033 27567268

