# Correction: Effectiveness of digital self-care device for at risk drinking problems: focus on individuals at risk for alcohol-related issues

**DOI:** 10.3389/fpsyt.2025.1644316

**Published:** 2025-06-30

**Authors:** Yong Chan Jeong, Yong Jin Kim, Sung Won Roh, Eun Seon Seo, Hong Seok Oh, In Suk Lee, Eun Ji Lee, Hyeon Ji Cho, Sang Kyu Lee

**Affiliations:** ^1^ College of Medicine, Hallym University, Chuncheon, Gangwon, Chuncheon, Republic of Korea; ^2^ Department of Social Welfare, Welfare and People Addiction Prevention Institute, Seoul, Seoul, Republic of Korea; ^3^ Department of Psychiatry, Hanyang University Seoul Hospital, Seoul, Seoul, Republic of Korea; ^4^ Department of Social Welfare, Integrated Addiction Management Support Center, Hwaseong, Gyeonggi, Hwaseong, Republic of Korea; ^5^ Department of Psychiatry, Konyang University Hospital, Daejeon, Daejeon, Republic of Korea; ^6^ Department of Nursing, Integrated Addiction Management Support Center, Suwon, Suwon, Republic of Korea; ^7^ Department of Counseling Psychology, Sahmyook University, Seoul, Republic of Korea; ^8^ Department of Psychiatry, Hallym University Medical Center, Chuncheon, Gangwon, Republic of Korea

**Keywords:** alcohol use disorder, digital self-care device, continuous days of sobriety, machine learning, ROC curve, multiple regression

In the published article, there was an error in the **Funding** statement.

“The author(s) declare that financial support was received for the research and/or publication of this article. This paper was supported by the Korea Institute of Industrial Technology Planning and Evaluation, funded by the Government of Korea in 2023 (No. RS-2022-KH125845) IRB No. 2023-04-002-0032. This research was supported by Hallym University Research Fund.”

The correct statement appears below:

“The author(s) declare that financial support was received for the research and/or publication of this article. This work was supported by a grant from the Korea Health Technology R&D Project through the Korea Health Industry Development Institute (KHIDI), funded by the Ministry of Health & Welfare, Republic of Korea (grant number: HI22C0707, RS-2022-KH125845).”

The authors apologize for this error and state that this does not change the scientific conclusions of the article in any way. The original article has been updated.

