# Correction: Effectiveness of digital self-care device for at risk drinking problems: focus on individuals at risk for alcohol-related issues

**DOI:** 10.3389/fpsyt.2026.1877781

**Published:** 2026-06-11

**Authors:** Yong Chan Jeong, Yong Jin Kim, Sungwon Roh, Eun Seon Seo, Hong Seok Oh, In Suk Lee, Eun Ji Lee, Hyeon Ji Cho, Sang-Kyu Lee

**Affiliations:** 1 College of Medicine, Hallym University, Chuncheon, Gangwon, Chuncheon, Republic of Korea; 2 Department of Social Welfare, Welfare and People Addiction Prevention Institute, Seoul, Seoul, Republic of Korea; 3 Department of Psychiatry, Hanyang University Seoul Hospital, Seoul, Seoul, Republic of Korea; 4 Department of Social Welfare, Integrated Addiction Management Support Center, Hwaseong, Gyeonggi, Hwaseong, Republic of Korea; 5 Department of Psychiatry, Konyang University Hospital, Daejeon, Daejeon, Republic of Korea; 6 Department of Nursing, Integrated Addiction Management Support Center, Suwon, Suwon, Republic of Korea; 7 Department of Counseling Psychology, Sahmyook University, Seoul, Republic of Korea; 8 Department of Psychiatry, Hallym University Medical Center, Chuncheon, Gangwon, Republic of Korea

**Keywords:** alcohol use disorder, digital self-care device, continuous days of sobriety, machine learning, ROC curve, multiple regression

Authors “Sungwon Roh and Sang-Kyu Lee” were erroneously spelled as “Sung Won Roh, Sang Kyu Lee”.

The funder “Hallym University Research Fund” was erroneously omitted. The correct funding statement is:

“The author(s) declare that financial support was received for the research and/or publication of this article. This work was supported by a grant from the Korea Health Technology R&D Project through the Korea Health Industry Development Institute (KHIDI), funded by the Ministry of Health & Welfare, Republic of Korea (grant number: HI22C0707, RS-2022-KH125845). This research was supported by Hallym University Research Fund”.

The original version of this article has been updated.

